# Drying Process of HPMC-Based Hard Capsules: Visual Experiment and Mathematical Modeling

**DOI:** 10.3390/gels9060463

**Published:** 2023-06-05

**Authors:** Chuqi He, Yucheng Yang, Mi Zhang, Kecheng Zhou, Yayan Huang, Na Zhang, Jing Ye, Moses Arowo, Bingde Zheng, Xueqin Zhang, Honghui Xu, Meitian Xiao

**Affiliations:** 1 Department of Chemical and Pharmaceutical Engineering, School of Chemical Engineering, Huaqiao University, Xiamen 361021, China; 2 Xiamen Engineering and Technological Research Center for Comprehensive Utilization of Marine Biological Resources, Xiamen 361021, China; 3 Department of Chemical Engineering and Chemistry, Eindhoven University of Technology, Het Kranenveld, 5600 MB Eindhoven, The Netherlands; 4 Department of Chemical & Process Engineering, Moi University, Nairobi 3900-30100, Kenya; 5 Zhejiang Honghui Capsule Co., Ltd., Shaoxing 312500, China

**Keywords:** plant-based polysaccharide gels, drying, LF-MRI, mathematical modeling, film

## Abstract

Using plant-based polysaccharide gels to produce hard capsules is a novel application of this technology in the medicinal field, which has garnered significant attention. However, the current manufacturing technology, particularly the drying process, limits its industrialization. The work herein employed an advanced measuring technique and a modified mathematical model to get more insight into the drying process of the capsule. Low field magnetic resonance imaging (LF-MRI) technique is adopted to reveal the distribution of moisture content in the capsule during drying. Furthermore, a modified mathematical model is developed by considering the dynamic variation of the effective moisture diffusivity (*D*_eff_) according to Fick’s second law, which enables accurate prediction of the moisture content of the capsule with a prediction accuracy of ±15%. The predicted *D*_eff_ ranges from 3 × 10^−10^ to 7 × 10^−10^ m^2^·s^−1^, which has an irregular variation with a time extension. Moreover, as temperature increases or relative humidity decreases, there is an increased acceleration of moisture diffusion. The work provides a fundamental understanding of the drying process of the plant-based polysaccharide gel, which is crucial for enhancing the industrial preparation of the HPMC-based hard capsules.

## 1. Introduction

Hard capsules have attracted considerable attention to the high utilization and stability of drugs [1]. Nevertheless, commercial pharmaceutical capsules are mainly made of gelatin and thus suffer several initial shortcomings, such as the cross-linking of gelatin and drug incompatibilities, the mammal origin of gelatin limitation, and the instability on exposure to high temperature and humidity [2,3,4]. Consequently, much attention has been paid to the development of plant-based polysaccharide hard capsules, which have a safer source of materials than gelatin-based capsules [5,6]. Thereinto, the capsules prepared by hydroxypropyl methylcellulose (HPMC) may offer an attractive alternative to gelatin-based capsules.

Hot-air drying plays an essential role in producing HPMC-based hard capsules. If drying conditions are improper, such as low relative humidity and high temperature, it leads to a variety of defects, such as blisters, warping, layer structure, and cracks, and hence, poor-quality products [7]. Moreover, drying is an energy-intensive operation, as it typically accounts for 20% to 25% of the energy requirement in industrial food production [8]. Therefore, it is crucial to establish a reasonable drying process that can yield high-quality capsules, minimize energy consumption, and reduce production costs. However, there is limited research on the drying process of the HPMC-based hard capsules. While the effects of drying conditions on the capsules have been discussed from a macro perspective in our previous study [7], a more comprehensive understanding of the drying process and its impact on moisture transportation still requires further investigation.

Mathematical modeling can enable a better understanding and prediction of the drying behavior and kinetics of HPMC-based hard capsules and thus help in optimizing the drying condition for industrial production. However, to the best of our knowledge, there are few studies on drying models for HPMC-based hard capsules. Literature suggests that the capsule shell can be considered a thin layer [9], an assumption that has been widely applied in some polymer and edible films. Drying mathematical models based on thin-layer have received excellent practicability and predictability [10,11]. Thin-layer drying models are categorized as theoretical, semi-theoretical, and empirical. Theoretical models are derived from Fick’s second law of diffusion and can theoretically predict drying behavior under various drying conditions. However, they lack fundamental parameters, limiting their application [12]. Empirical models, on the other hand, can give perfect results in predicting drying behavior owing to their dependence on experimental dates. However, they are still not able to reveal deeper and more detailed information about the drying process [13]. Semi-theoretical drying models such as the Page, Lewies, and Logarithmic models are also derived from Fick’s second law. These models make fewer assumptions and require a small amount of experimental data [14]. Consequently, they are widely applied in predicting thin-layer drying behaviors, including onion slices [15], raw mango slices [16], and pullulan–alginate-based edible films [17].

The effective moisture diffusivity (*D*_eff_) is a fundamental parameter in drying models. Nevertheless, given the limitations of experimental techniques and the reliance on simplified calculations, *D*_eff_ has often been assumed to be a constant value in many studies [11,18,19]. In our previous work [7], we obtained a constant value of *D*_eff_ for each drying process. Some studies have suggested that variable *D*_eff_ is more precise than constant *D*_eff_ in describing the water absorption of composite materials and chitosan film [20]. It has been verified that *D*_eff_ varies with internal material conditions such as moisture content, temperature, and structure [21,22]. Due to the unfeasible theoretical prediction of *D*_eff_ in complex materials, the value of *D*_eff_ is usually obtained from experimental results through computer optimization and the slopes method [23]. The computer optimization method can give *D*_eff_ at different grid point and time step through the iteration and the discretization [9]. Experimentation is a direct method to study the drying process of the HPMC-based hard capsules, and accurate experimental data are essential in developing a reasonable drying model. The experimental drying data should mainly be obtained by weighing the sample at a certain time interval, which hardly conforms with the requirements of high-quality production and the development of proper mathematical models. However, modern technologies such as microcosmic techniques for detecting moisture have been gradually adopted to analyze the drying process in food, agriculture, pharmaceutics, architecture, and other related industries [24,25,26].

Low-field magnetic resonance imaging (LF-MRI) is a rapid non-destructive detection technology that has been used in quantitative analysis of the distribution of moisture during drying owing to its unique advantages of high sensitivity, strong pertinence, and short detection time [27]. There are three types of ^1^H images: *T*_1_, *T*_2_, and proton density-weighted images. The proton density-weighted images reflect the density and content of hydrogen protons. The technique has been successfully used to analyze the variation in moisture distribution and model the spatial moisture distribution during drying [28,29]. Therefore, LF-MRI is supposedly a novel approach that can provide detailed information on the drying process of the thin layer.

The study herein employed the LF-MRI technique to analyze the effect of different drying conditions on the moisture content and distribution of HPMC-based hard capsules. Additionally, a reasonable drying mathematical model with a variable *D*_eff_ is also developed based on Fick’s second law. The predicted results are then compared with those of the experiment for conformity. The aim of this study is to provide a deeper insight into the drying process of HPMC-based hard capsules.

## 2. Results and Discussion

### 2.1. LF-MRI Analysis

**Figure 1:**
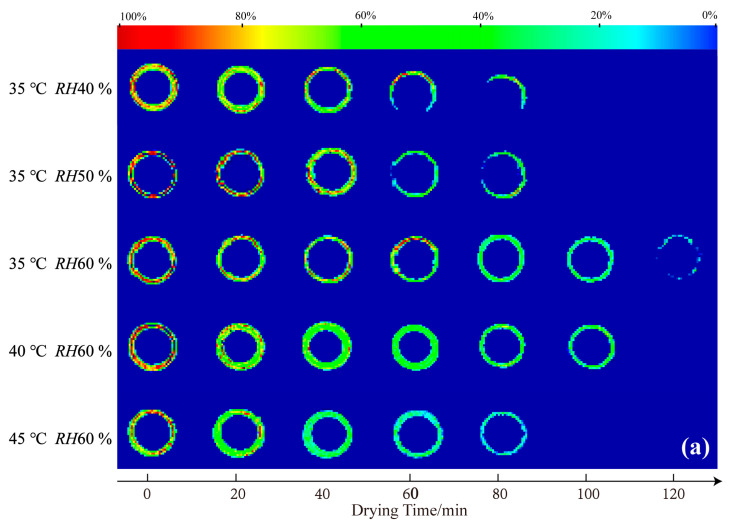

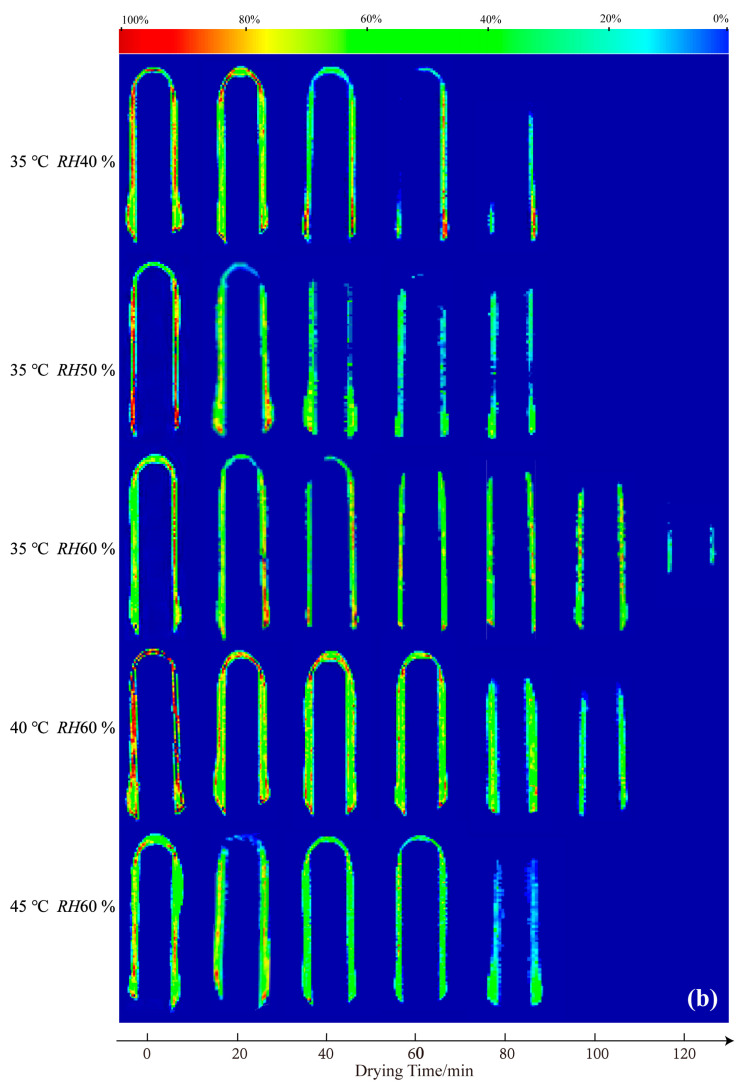
Cross-sectional (**a**) and vertical-sectional (**b**) proton density-weighted images of the HPMC-based hard capsules under different drying conditions.

LF-MRI can show the spatial distribution of protons in the capsule shell at different stages during the drying process. Figure 1 shows cross-sectional (a) and vertical-sectional (b) MRI images of the HPMC-based hard capsules during the drying process. The color of the cross-sectional and vertical-sectional MRI images correspond to each other. Different colors in the images indicate different moisture percentages and reflect the moisture content in the capsule shell. The moisture content of the capsule gradually decreases from red to green to blue in the color strip.

It is evident that the initial colors in the middle layer of all capsules are almost red, an indication that the initial moisture content under different drying conditions is the same. As drying time increases, the moisture percentage continuously decreases (green area increases), indicating a decreasing trend of the moisture content of the capsule with time. After 60 min, the moisture percentage distribution in the cross-sectional capsule shell gradually becomes more uniform, an indication of even moisture distribution and that the inner moisture of the capsule is transferring to the surface of the capsule. At the end of the drying process, the contour of the capsule becomes more blurred, and the moisture percentage equals 0% after 80 min, indicating that the majority of moisture has evaporated and that only the bound water possibly leaves [28,30]. Meanwhile, in the vertical sectional MRI images, it is obvious that the moisture percentage signal first disappeared at the elbow of the capsule, attributed to the thinnest in this part and finally at the broadside part. The signal lastly disappears at the bottom of the capsule. This is probably because the thickness of the bottom is larger than other parts of the capsule, attributed to the effect of gravity, which results in the collection of some mixture solution and calcification solution at the bottom part. Noticeably, slight variations in the overall color of the capsules are observed among the slices under different drying conditions at 0 min. This phenomenon could be attributed to the positioning of LF-MRI, the selection of LF-MRI images, and potential errors in the preparation process. However, the results of quantitative detection verify that the initial moisture content of each capsule is consistent throughout, with an average deviation of less than 5%.

The moisture content rapidly decreases, and the color variation becomes more obvious at a lower relative humidity and higher temperature. At 35 °C, the moisture content at *RH*40 % or *RH*50 % rapidly decreases, indicating fast moisture disappearance and high average effective moisture diffusivity (*D*_eff-avg_). This situation may cause the evaporation of moisture from the outside surface before the inside moisture supply to the outside surface, resulting in a loose network structure and cracks. The results herein are consistent with those of increasing temperature or decreasing relative humidity to increase the drying rate. LF-MRI, therefore, provides a novel approach to obtaining detailed information on the effect of various drying conditions on moisture content and distribution of HPMC-based hard capsules.

### 2.2. Effect of Various Drying Conditions on Average Moisture Content and D_eff_ of HPMC-Based Hard Capsules

**Figure 2:**
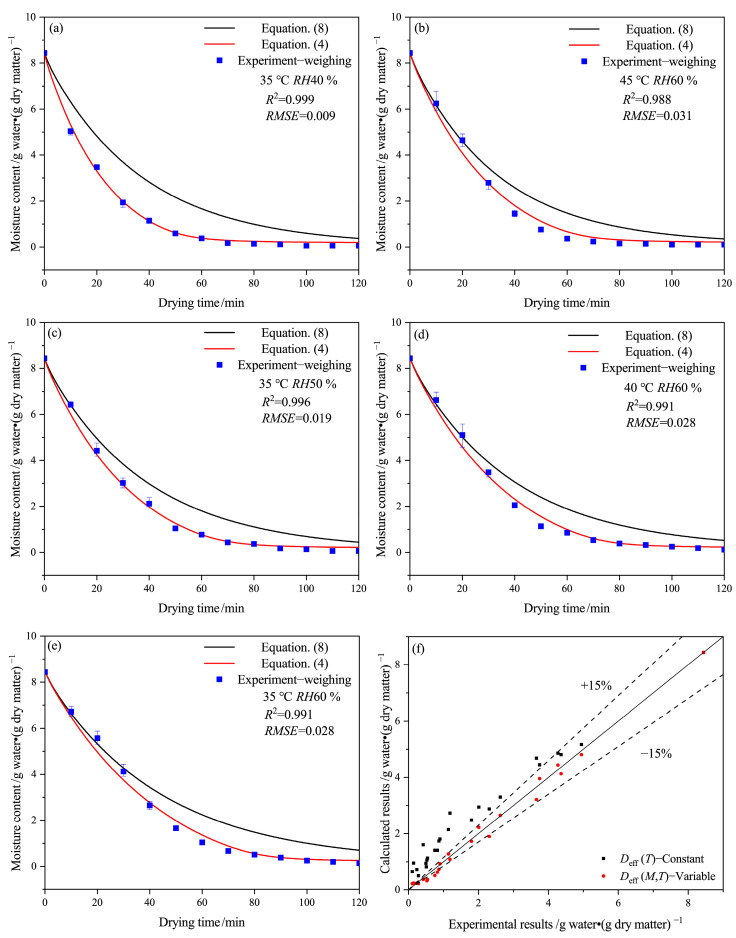
Drying curves at variable or constant *D*_eff_ of the HPMC-based hard capsules under different drying conditions: (**a**) 35 °C *RH*40 %, (**b**) 45 °C *RH*60 %, (**c**) 35 °C *RH*50 %, (**d**) 40 °C *RH*60 %, (**e**) 35 °C *RH*60 % and (**f**) comparison between experimental and calculated results.

Figure 2 illustrates the effect of drying conditions on the moisture content of HPMC-based hard capsules based on the experiments and drying mathematical models with variable (Equation (4)) or constant (Equation (8)) *D*_eff_, as well as a comparison between these results. It is evident that the calculated results at variable *D*_eff_ are in better agreement with the experimental results obtained by the weighing method than those using constant *D*_eff_. Furthermore, the difference between the two calculated results becomes more significant with time. The moisture content decreases rapidly from 8.44 to 0.5 g water·(g dry matter)^−1^ with time until the 80th minute and then slowly decreases from 0.5 g water·(g dry matter)^−1^ to the equilibrium moisture content. The drying rate is slower at lower temperatures or higher relative humidity, and it takes longer to reach the equilibrium moisture content. There are some deviations between the calculated results of Equation (4) and the experimental results before 30 min, with relative errors under 15%. This deviation may be due to the assumptions of uniform temperature distribution and temperature equivalence of hot air and the sample since there may be a temperature difference between them at the early stage of the drying process [31]. As the drying time increases, the prediction results of Equation (4) become more accurate, indicating that the changes in the internal structure of capsules caused by a decrease in moisture content directly affect the effective moisture diffusivity. Figure 2f shows that the experimental value for the average moisture content of the capsule has good agreement with the ones calculated with Equation (4), with an error of around 15%.

**Figure 3:**
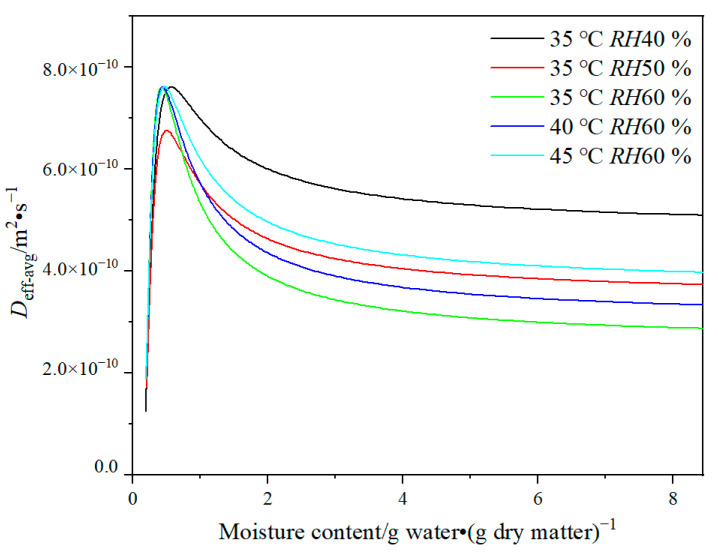
Plot of *D*_eff-avg_ versus moisture content of HPMC-based hard capsules under different drying conditions.

The results indicate that the variability of *D*_eff_ values in the drying model is a critical parameter to achieve an accurate forecast for the HPMC-based hard capsules. The effects of various drying conditions and moisture content on *D*_eff-avg_ values are shown in Figure 3. It is evident that *D*_eff-avg_ ranges from 3 × 10^−10^ to 7 × 10^−10^ m^2^·s^−1^. It is obvious that *D*_eff-avg_ increases slowly as the moisture content decreases from 8.44 to 2 g water·(g dry matter)^−1^, and then rapidly increases under moisture content ranging from 2 to 0.5 g water·(g dry matter)^−1^, and finally decreases under moisture content ranging from 0.5 g water·(g dry matter)^−1^ to the equilibrium moisture content. The value and tendency of *D*_eff-avg_ are similar to that of porous food material containing many positions for attraction of water molecules [32], such as bio-polysaccharide membrane [33,34], fruit, and seed [21,35]. In general, the irregular variation of *D*_eff-avg_ is related to the change in the drying mechanism [36]. The rising tendency of *D*_eff-avg_ may be attributed to the fact that during the early stages of drying, liquid diffusion is the primary drying mechanism, followed by vapor diffusion as more pores and cracks form owing to the decreasing moisture content [33]. Thus, moisture transport is facilitated. At the end of the drying process, the moisture content of the capsule shell is close to the equilibrium moisture content, and thus *D*_eff-avg_ rapidly reduces. This observation is due to the low moisture content, which results in only bond water being present and having a strong connection with the molecular structure of the polymer [33].

At moisture content ranging from 2 to 8.44 g water·(g dry matter)^−1^, *D*_eff-avg_ decreases as temperature decreases and relative humidity increases, leading to a slow drying rate. The trend suggests that at higher temperatures or lower relative humidity, there is an increased acceleration of moisture diffusion of internal moisture, making the moisture easier to evaporate [37]. *D*_eff-avg_ is minimum at 35 °C and *RH*60 % but maximum at 35 °C and *RH*40 %, with its value higher than that at 45 °C and *RH*60 %. The phenomenon can be attributed to the rapid evaporation of moisture connected to polysaccharide molecules, weak molecular network connections, and the loose form, resulting in cracks and layer structure of the capsule shell. It has been proven that improper drying conditions can lead to macro characteristics of capsules that are considered unqualified [7]. In this case, the drying mechanism may change from liquid diffusion to vapor diffusion. It also suggests that lower relative humidity may have a greater impact on moisture diffusion than higher temperature during the drying process.

### 2.3. Effect of Drying Conditions on Moisture Content and D_eff_ at Each Layer of the Capsule Shell

**Figure 4:**
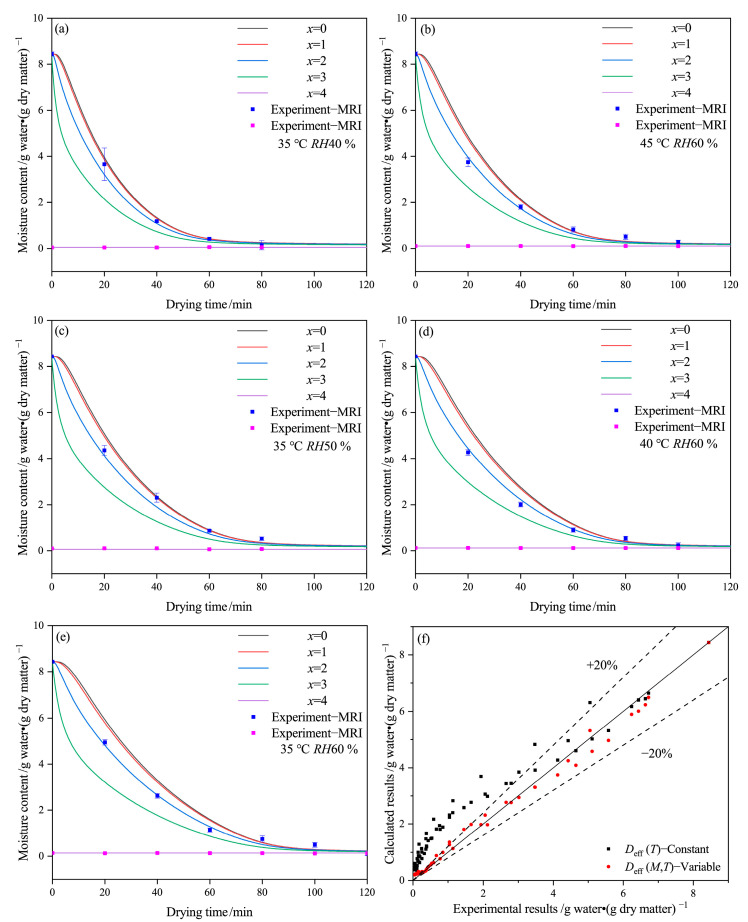
Drying curve obtained by proton density-weighted images and modeling of the HPMC-based hard capsules under different drying conditions: (**a**) 35 °C *RH*40 %, (**b**) 45 °C *RH*60 %, (**c**) 35 °C *RH*50 %, (**d**) 40 °C *RH*60 %, (**e**) 35 °C *RH*60 %, and (**f**) comparison between experimental and calculated results.

Figure 4 shows the experimental results obtained by LF-MRI technology and the calculated results for each layer of the capsule, along with a comparison between these results. The data from the outside layer of the MRI images represent the equilibrium water, while only the experimental data from the middle layer of MRI images are shown due to the boundary effect. The simulation results at the outside surface of the capsule (*x* = 4) remain constant due to the initial condition of Equation (4). The numerical moisture content (*x* = 2) shows considerable agreement with the data of the middle layer obtained by MRI images. However, due to device limitations and the thickness of the capsule shell, MRI images only have three to five pixels, which can affect the accuracy of selected layers and data of MRI images. As a result, the moisture contents obtained by MRI images deviate somewhat from the numerical results, which can affect the accurate fitting of this model.

Figure 4 shows that the moisture content mainly exists in the inside layers (*x* = 0 to *x* = 2) of the capsule. Under the same drying condition and time, the moisture content gradually decreases from the inside to the outside layers (*x* = 0 to *x* = 4), and the outside layer (*x* = 3) dries faster than the inside layers. Based on the calculated results, the main drying mechanism of the capsule is that moisture transfers from the inside layer (*x* = 0) to the outside layer (*x* = 4) and then evaporates into the environment. This observation is consistent with the experimental results from LF-MRI technology, which usually reaches equilibrium at first.

The drying curves in the *x* = 0 and *x* = 1 layers are relatively close, particularly at 35 °C and *RH* 40% or 45 °C and *RH* 60%. However, the gap between the *x* = 2 and *x* = 3 curves increases as the temperature decreases or the relative humidity increases, indicating that moisture concentration differences become larger at lower temperatures and higher relative humidity. The increased gap may result from a reduction in the transfer rate of moisture from the inside layer to the outside layer, which leads to a reduced average value of *D*_eff-avg_. Figure 4f provides evidence that Equation (4) can be used not only to estimate the mean moisture content but also to predict the moisture content at each layer of the capsule, with an error of approximately 20%.

**Figure 5:**
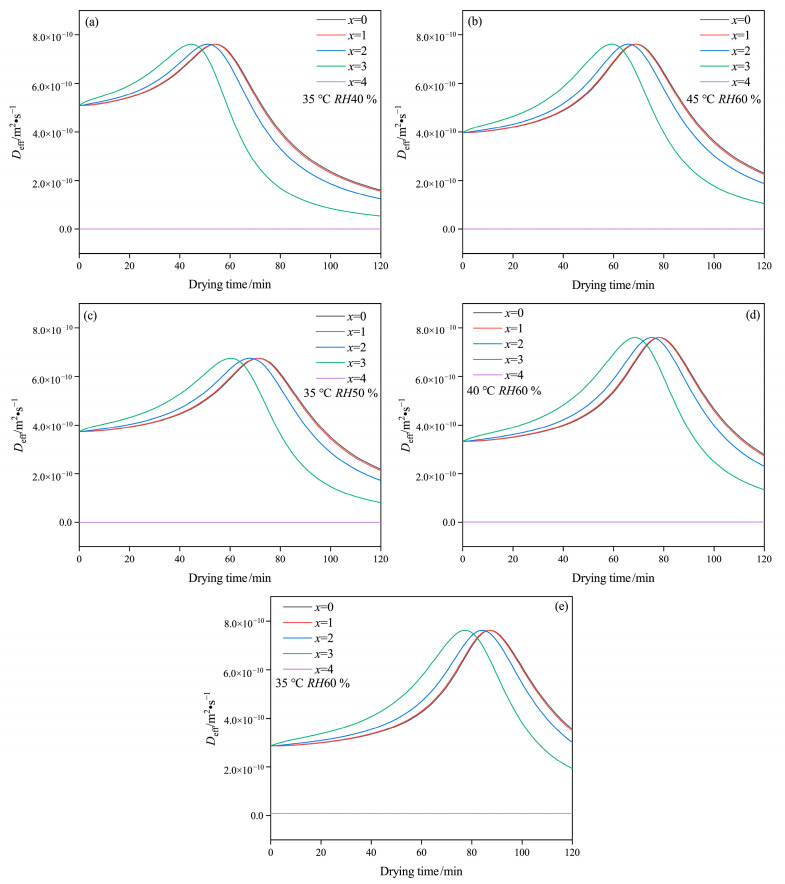
Variation of *D*_eff_ of HPMC-based hard capsules under different drying conditions: (**a**) 35 °C *RH*40 %, (**b**) 45 °C *RH*60 %, (**c**) 35 °C *RH*50 %, (**d**) 40 °C *RH*60 %, (**e**) 35 °C *RH*60 %.

Figure 5 presents the effect of drying conditions on *D*_eff_ at each layer, with consistent variation patterns throughout the drying period. It has been noted that *D*_eff_ relates to moisture content during the drying process. The polysaccharide structure of the capsule affects the connection between molecules and moisture, and the transfer of moisture may also affect the physical structure of the capsule [38]. At the initial drying stage, just before *D*_eff_ reaches its maximum, the value of *D*_eff_ at the outside layer is larger than that at the inside layer. However, the comparison result becomes the opposite when the value of *D*_eff_ reduces. This can be attributed to the fact that at the early stage of drying, the moisture on the outside layer, which has a larger contact area with hot air, easily evaporates to the hot air, while the moisture on the inside layer should mainly transfer onto the outside layer via liquid diffusion. At the late drying stage, the content of water connected to polysaccharide molecules may become smaller from *x* = 0 to *x* = 4, leading to a lower *D*_eff_. In this case, the small amounts of bound water are likely to evaporate, resulting in a slow drying rate at the end of the drying process.

The drying curves in the layer of *x* = 0 and *x* = 1 are relatively close, while the gap gradually increases between *x* = 2 and *x* = 3 with a decrease in temperature and an increase in relative humidity. Furthermore, as temperature increases or relative humidity decreases, the maximum in the lines of *D*_eff_ versus drying time move to the left at drying time range from 100 to 60 min. This result likely suggests that moisture associated with molecules diffuses and evaporates at a faster rate and that even bound water content may decrease more quickly during rapid drying. Thus, as drying time is extended, the weakening of the network structure due to the rapid drying of bound water may result in cracks and layer structure formation under unsuitable drying conditions, such as low relative humidity and high temperature. Consequently, these conditions can result in poor performances, including increased brittleness and reduced storage stability.

## 3. Conclusions

The study employs the LF-MRI technique to analyze the drying process of the HPMC-based hard capsules under various drying conditions. The moisture content decreases at a faster rate under lower relative humidity or higher temperature. Cross-sectional and vertical-sectional MRI images of the capsule indicate that moisture distribution continuously decreases and gradually becomes uniform after 60 min of the drying process. Due to the configuration and preparation process of the capsule, moisture is inclined to first disappear at the elbow, then at the broadside part, and finally at the bottom of the capsule. Additionally, a reasonable drying mathematical model with a variable *D*_eff_ is developed based on Fick’s second law. The results show that the drying process is better described by the model with variable *D*_eff_ than that of a constant value, and the calculated *D*_eff-avg_ exhibits irregular variation within the range of 3 × 10^−10^ to 7 × 10^−10^ m^2^·s^−1^. Moreover, this model is able to simulate the moisture content and *D*_eff_ at each layer. The results show that with the extension of drying time, the moisture content of the capsule continually decreases from the inside layer (*x* = 0) to the outside layer (*x* = 4) under the same drying condition and time. The calculated results of each layer and the whole capsule during the drying process support that the drying mechanism changes and bound water prefer to be partially removed as the temperature increases or relative humidity decreases, which are the results of the layer structure and crack generation. Thus, according to previous studies, combined with drying quality and efficiency, drying conditions at 35 °C and *RH*60 % are found to be suitable for the HPMC-based hard capsules.

## 4. Materials and Methods

### 4.1. Materials

Commercially available food-grade HPMC (HT-E5), calcium chloride, and potassium citrate were sourced from Shandong Ruitai Co., Ltd. (Feicheng, China), while xanthan gum and gellan gum were purchased from Shanghai Macklin Biochemical Co., Ltd. (Shanghai, China). Analytical reagent sodium alginate was also supplied by Shanghai Macklin Biochemical Co., Ltd. (China). Distilled water was used in the preparation of all solutions used in the experiment.

### 4.2. Sample Preparation

A solution was successively prepared by dissolving potassium citrate (0.3%, *w/w*), gellan gum (0.4%, *w/w*), xanthan gum (0.4%, *w/w*), and sodium alginate (0.7%, *w/w*), and HPMC (9%, *w/w*) in distilled water at a moderate agitation speed of 480 r·min^−1^. The solution was then heated to 80 °C and then maintained for 40 min to ensure that the materials completely dissolved. The solution mixture was then cooled to 60 °C for 4 h to remove bubbles. The HPMC-based hard capsules were prepared using the conventional method of dipping preparation whole mold into the solution [39,40] and then immediately immersing the formed capsules into a calcium chloride solution for 20 s to achieve enteric properties [7,41]. Finally, the capsules were dried in a dryer at a constant temperature and relative humidity. At each operating condition of the drying process, 15 capsules were prepared for the following detection measurement.

### 4.3. Drying Experiment

Based on our previous works [7,42] and considering production capacity, the capsules prepared on the mold pins were uniformly arranged on the shelf of a dryer, where they were subjected to a range of drying temperatures (i.e., 35, 40, and 45 °C) while maintaining a fixed relative humidity of 60%. Additionally, the capsules were exposed to varying relative humidity levels (i.e., 40, 50, and 60%) while keeping the temperature fixed at 35 °C. Hot air (2 m·s^−1^) entered the dryer and flowed upwards across the capsule samples. During each operating condition of the drying process, samples were randomly selected from the prepared capsules in the dryer. These samples were immediately weighed and subsequently scanned by LF-MRI at an interval of 20 min until a constant weight or weak signal was achieved. Typically, this required the detection of 5–7 capsules. LF-MRI measurement for both the cross-section and vertical section took 6 min each. The detected capsules were not placed in the oven for the next detection. The detected samples were finally placed in the dryer at 105 °C until constant mass weight to obtain the dry matter mass. The data from the drying experiments were recorded in triplicate, with an average experimental error of less than 10%.

### 4.4. LF-MRI Measurements

Each HPMC-based hard capsule was carefully transferred into a nuclear magnetic tube and scanned using a magnetic resonance imaging analyzer (NMI20-060H-Ⅰ, Suzhou Niumag Analytical Instrument Co., Suzhou, China). The analyzer operated at a resonance frequency of 21 MHz at 32 °C (the magnet temperature). The spin echo sequence was used to reconstruct proton density-weighted images in two dimensions, representing the cross section and vertical section of the capsule shell. The signal intensity (*I*/a.u) of the proton density-weighted images can be determined as follows [43]:

(1) $$I=k\rho_{H}\left[1-exp\left(-T_{R}/T_{1}\right)\right]exp\left(-T_{E}/T_{2}\right)$$

where *k* is the proportionality constant, *ρ*_H_ is the density of the hydrogen nuclei (m^−3^), *T*_R_ is repetition time (ms), *T*_E_ is echo-time (ms), *T*_1_ is longitudinal relaxation time (ms), *T*_2_ is transverse relaxation time (ms).

**Figure 6:**
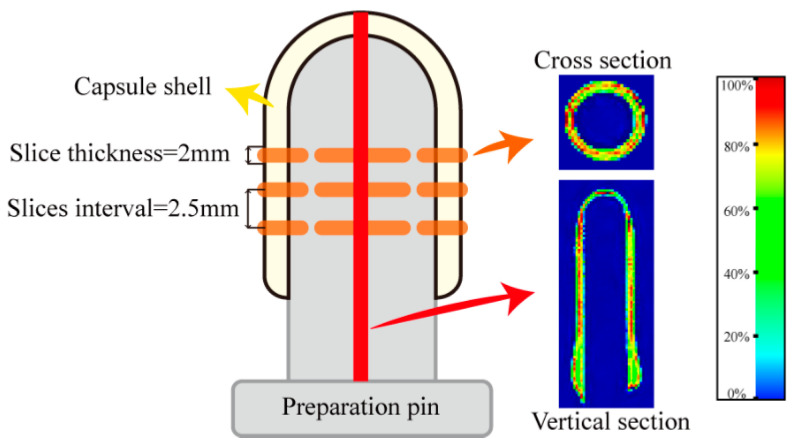
Schematic diagram of the position in the cross-section and vertical section of MRI images of the HPMC-based hard capsules and the standard color strip.

The main parameters of the Numag analyzer were set as follows: FOV (Field-of-view) = 100 × 100 mm, slice thickness = 2 mm, *T*_R_ = 2000.00 ms, *T*_E_ = 18.125 ms. In the cross-section of the capsule shell, the number of slices was three. Cross-sectional MRI images were obtained in the position excluding the elbow, while the vertical section was cut from the symmetry line of the capsule, as shown in Figure 6. The MRI images were acquired with an average based on two scan repetitions. Unified mapping and pseudocolor processing were performed to improve the quality of the images.

Since the signal intensity of the proton density-weighted image has a linear relation with the moisture content of the material [44,45,46], the initial signal intensity in the capsule (*t* = 0 min) was set to 100% on the standard color strip. When the moisture content in the capsule reached equilibrium (i.e., the signal intensity in the capsule was similar to the blank zone), the final signal intensity in the capsule was set to 0%. The moisture percentage (%) of the standard color strip is linearly related to its signal value, as shown in Figure 6. The moisture percentage of the MRI images can be calculated using the following equation:

(2) $$\mathrm{Moisture}\;\mathrm{percentage}\left(A,t\right)=\frac{\sum_{0}^{A}I\left(x,y,t\right)/N-\sum_{0}^{A}I\left(x,y,t=\mathrm{Final}\right)/N}{\sum_{0}^{A}I\left(x,y,t=0\right)/N-\;I\sum_{0}^{A}I\left(x,y,t=\mathrm{Final}\right)/N}\times 100\%$$

where *I* (*x*, *y*, *t*) is the local signal intensity of the middle layer in the MRI images of the capsule under different drying conditions (a.u), A is the zone of the middle layer in the MRI images of the capsule, *N* is the number of pixels in the zone, and *t* is the time (s).

### 4.5. Modeling the Drying Curve

**Figure 7:**
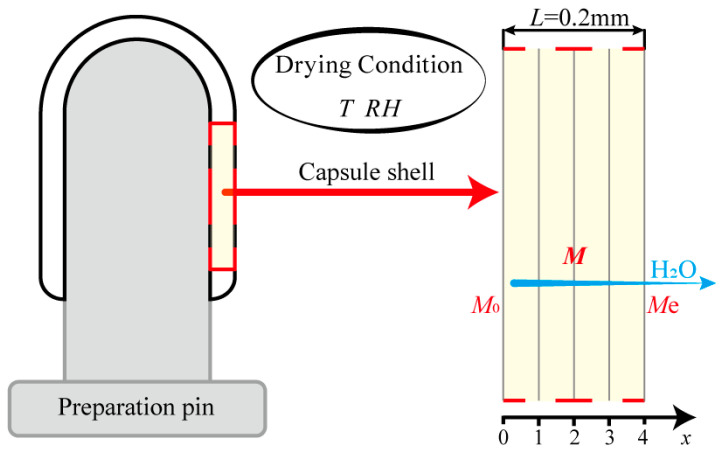
Schematic diagram of the HPMC-based hard capsules during the drying process.

Figure 7 shows a schematic diagram of the HPMC-based hard capsules during drying. Since the inside surface of the capsule is tightly closed to the preparation pin, moisture in the capsule is considered to be transferred only from the inside to the outside surface and finally evaporated into the atmosphere. A previous study of HPMC-based hard capsules [7] has shown that the drying process of the capsule occurs in the falling-rate period when the relative humidity is less than 60%. Moreover, due to the particular thin layer and structure of the capsule, several assumptions have been made [13,47,48], including:

(1) The drying process is assumed to be one-dimensional mass transport;
(2) The initial moisture distribution of the capsule shell is considered to be uniform;
(3) The shrinkage of the capsule is ignored;
(4) The moisture content on the surface of the thin layer reaches equilibrium instantaneously [12,49];
(5) The temperature of the capsule shell is assumed to be the same as the hot-air temperature.

As the drying time extends, moisture diffusion becomes the dominant diffusion mechanism, including surface diffusion on the pore surfaces, liquid diffusion, or vapor diffusion due to differences in moisture concentration [50,51]. Hence the moisture diffusion process is described by Fick’s second law of diffusion as follows [52]:

(3) $$\frac{\partial M}{\partial t}=\nabla \left(D_{\mathrm{eff}}\;\cdot \;\nabla M\right)$$

where *M* is the local moisture content on a dry basis (g water·(g dry matter)^−1^). *D*_eff_ is the effective moisture diffusivity (m^2^·s^−1^), which can vary considerably with the moisture inside the material and drying conditions [53,54]. Then, Equation (3) can be written as follows:

(4) $$\frac{\partial M\left(x,t\right)}{\partial t}=\frac{\partial }{\partial x}\left[D_{\mathrm{eff}}\left(M,T\right)\frac{\partial M\left(x,t\right)}{\partial x}\right],\;0<x<L,t>0$$

where *x* is diffusion path (m), *T* is the drying temperature, *M* (*x*, *t*) is the local moisture content on a dry basis (g water·(g dry matter)^−1^), related to *x* and *t*, and *L* is the thickness of the sample, with a value of 0.0002 m.

The Initial condition is written as follows:

$$t=0,\;0\leq x\leq L,\;M=M_{0}$$

Boundary conditions are written as follows:

$$t\geq 0,\;x=L,\;M=M_{e}$$

$$t\geq 0,\;x=0,\frac{\partial M}{\partial x}=0$$

To numerically solve Equation (4) with the initial and boundary conditions, the explicit finite difference method was used in order to calculate moisture content and *D*_eff_. Firstly, the capsule shell is divided into *Z* layers (where *Z* = 4) of thickness, ∆l=L/Z. It is assumed that *x* is the horizontal grid index, and *j* is the time grid index. The time step ∆*t* needed to be validated to ensure numerical stability. Then, the respective location of the intermediate grid node between layers is given by $L_{x}=x∆l$ and respective time steps were  t=j∆t. Lastly, the moisture content is calculated by discretization of Equation (4). The discretization process is as follows [49]:

(5) $$\frac{M_{0}^{t+1}}{∆t}=\frac{M_{0}^{t}}{∆t}+\frac{6D_{\mathrm{eff}}{|}_{0}^{t}}{{\left(∆l\right)}^{2}}\left(M_{1}^{t}-M_{0}^{t}\right)\;and\;∆t\leq \frac{1}{6}\frac{{\left(∆l\right)}^{2}}{D_{\mathrm{eff}}{|}_{0}^{t}},\;x=0$$

(6) $$\begin{array}{c} \frac{M_{x}^{t+1}}{∆t}=M_{x}^{t}\left(\frac{1}{∆t}-\frac{2D_{\mathrm{eff}}{|}_{x}^{t}}{{\left(∆l\right)}^{2}}\right)+\frac{M_{x+1}^{t}-M_{x-1}^{t}}{2∆l}\left(\frac{2D_{\mathrm{eff}}{|}_{x}^{t}}{x∆l}+\frac{D_{\mathrm{eff}}{|}_{x+1}^{t}-D_{\mathrm{eff}}{|}_{x-1}^{t}}{2∆l}\right)\\ \;\;\;\;\;\;\;\;\;\;\;\;\;\;\;\;\;\;\;\;\;\;\;\;\;\;\;\;\;\;\;\;\;\;\;\;\;\;\;\;\;\;\;\;+D_{\mathrm{eff}}{|}_{x}^{t}\frac{M_{x+1}^{t}-M_{x-1}^{t}}{{\left(∆l\right)}^{2}}and∆t\leq \frac{1}{2}\frac{{\left(∆l\right)}^{2}}{D_{\mathrm{eff}}{|}_{x}^{t}},\;0<x<L \end{array}$$

and *D*_eff_ is then calculated at each grid point *x* and time step *t* as follows [49]:

(7) $$D_{\mathrm{eff}}\left(M,T\right){|}_{x}^{t}=D_{o}exp\left[\frac{-E_{a}}{RT}+\frac{a}{M_{x}^{t}}-\frac{b}{{\left(M_{x}^{t}\right)}^{2}}\right]$$

where *E*_a_ is the activation energy (J·mol^−1^), *R* is the universal gas constant (8.3145 J·mol^−1^·K^−1^), a and b are model parameters, *D*_0_ is the Arrhenius factor that is generally defined as the reference diffusion coefficient at infinitely high temperature.

To compare the difference between variable and constant *D*_eff_, Equation (4) is written with a constant value of *D*_eff_ as follows [48,52]:

(8) $$\frac{\partial M\left(x,t\right)}{\partial t}=D_{\mathrm{eff}}\left(T\right)\frac{\partial^{2}M\left(x,t\right)}{\partial x^{2}},\;0<x<L,t>0$$

The solution of Equation (8) is also calculated by Equations (5) and (6).

The mean value of *D*_eff-avg_ is calculated from the numerical integration of the local values as follows:

(9) $$D_{\mathrm{eff}-\mathrm{avg}}\left(M,\;T\right){|}_{\;}^{t}=\frac{\int D_{\mathrm{eff}}\left(M,T\right){|}_{x}^{t}dM\left(x,t\right)}{\int dM\left(x,t\right)}$$

### 4.6. Data Analysis

Mathematical modeling of the drying process usually requires the use of statistical methods of regression and correlation analysis. The determination coefficient (*R*^2^) and root mean square error (*RMSE*) were used to evaluate the accuracy of the predicted model parameters

(10) $$R^{2}=1-\frac{\sum_{x=1}^{Z}{\left(M_{pre,x}-M_{exp,x}\right)}^{2}}{\sum_{x=1}^{Z}{\left(M_{pre,mean}-M_{exp,x}\right)}^{2}}$$

(11) $$RMSE={\left[\frac{\sum_{x=1}^{Z}{\left(M_{pre,x}-M_{exp,x}\right)}^{2}}{Z}\right]}^{\frac{1}{2}},\;x=1,2,3,\ldots \ldots ,Z$$

where *M_exp,x_* represents the experimental moisture content found in measurement, *M_pre,x_* represents predicted moisture content for this measurement, and *M_pre,mean_* represents the average of predicted moisture content for this measurement. *Z* is the number of points. A higher *R*^2^ and lower *RMSE* values indicate better goodness of fit.

The calculation process of the estimated parameter of *D*_eff_ is presented in Appendix A. The calculation results were obtained using the commercial software—MATLAB R2019b (The MathWorks Inc., Natick, MA, USA). The analysis data of experimental and signal intensity of MRI images were obtained using the Statistical Analysis System in Excel (Microsoft^®^, Redmond, WA, USA).

## Figures and Tables

**Figure A1:**
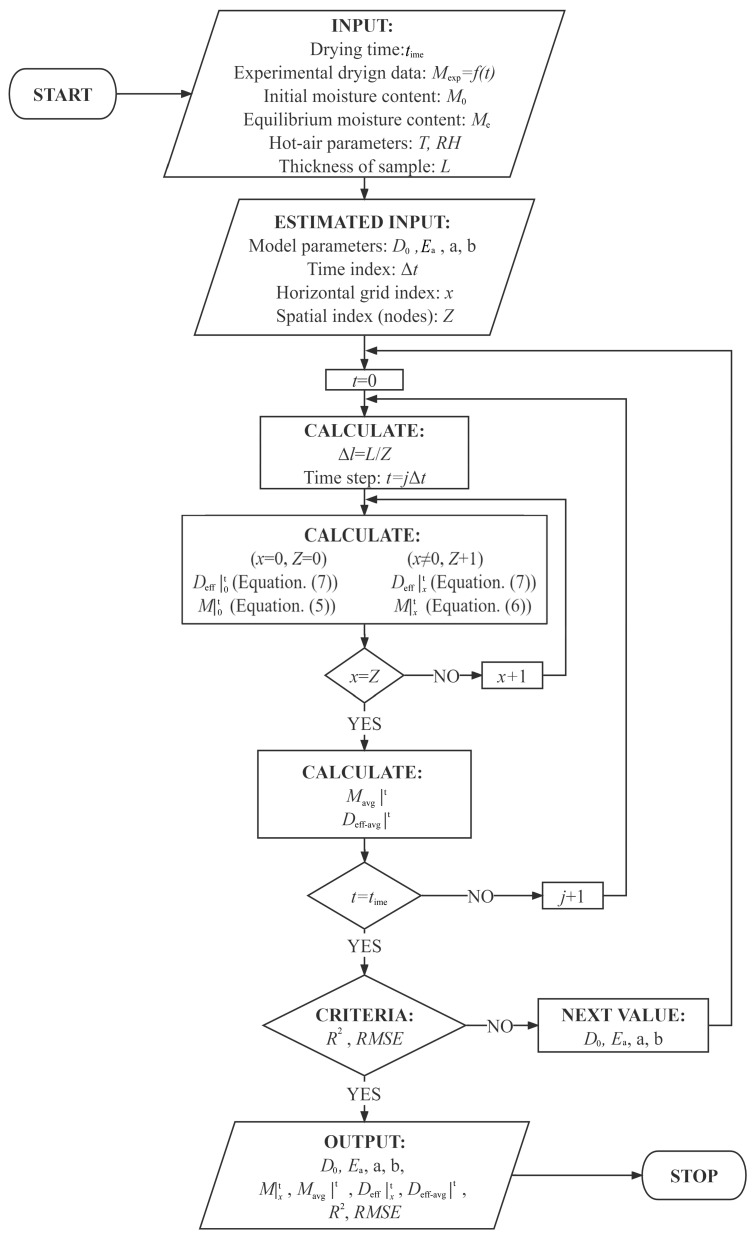
Schematic flowchart for calculating estimated parameters of *D*_eff_ of the HPMC-based hard capsules.

## Data Availability

The data used to support the findings of this study are available from the corresponding author upon request.

## Nomenclature

A	Zone of the middle layer in the MRI images of the capsule
*a*	Estimated parameter of Equation (7)
*b*	Estimated parameter of Equation (7)
*D*_eff_	Effective moisture diffusivity, m^2^·s^−1^
*D*_eff-avg_	Average effective moisture diffusivity, m^2^·s^−1^
*D*_0_	Arrhenius factor, m^2^·s^−1^
*E*_a_	Activation energy, kJ·mol^−1^
*k*	Proportionality constant
*L*	Thickness of capsule shell, mm
*M*	Moisture content, g water·(g dry matter)^−1^
*M*_e_	Equilibrium moisture content, g water·(g dry matter)^−1^
*M*_0_	Initial moisture content, g water·(g dry matter)^−1^
*M*_exp,x_	Experimental moisture content found in measurement, g water·(g dry matter)^−1^
*M*_pre,x_	Predicted moisture content for this measurement, g water·(g dry matter)^−1^
*M*_pre,mean_	Average of predicted moisture content for this measurement, g water·(g dry matter)^−1^
*N*	Number of pixels in the zone
*R*	Universal gas constant, J·mol^−1^·K^−1^
*R* ^2^	Coefficient of determination
*RH*	Relative humidity of hot air, %
*RMSE*	Root mean square error
*T*	Drying temperature of hot air, ℃
*T*_E_	Echo-time, ms
*T*_R_	Repetition time, ms
*T*_1_	Longitudinal relaxation time, ms
*T*_2_	Transverse relaxation time, ms
*t*	Time, s
*x*	Diffusion path, m
*Z*	Number of layers taken in discretization or the number of points
*ρ*_H_	Density of the hydrogen nuclei, m^−3^

## Appendix A

The schematic calculation process of estimated parameters of *D*_eff_ (a, b, *D*_0_, and *E*_a_) is presented in Figure A1. The capsule shell has meshed for iterative calculation. The results in *t* = 0 min, from *x* = 0 to *x* = *Z*, are firstly calculated, then is that in *t* min. The estimated parameters will reset according to the criteria during the calculation process.

Calculating by Equations (4)–(8), the estimated parameters of *D*_eff_ are presented in Table A1. It is evident that parameters a and b decreased as the temperature increased and relative humidity decreased. Furthermore, the relation between drying conditions (temperature (*T*) or relative humidity (*RH*)) and a or b is likely to be linear; this relationship between estimated parameters and drying conditions is shown as follows:

$$a=0.5520-0.0240\times T+0.02\times RH,\;R^{2}=0.9754$$

$$b=0.1741-0.0036\times T+0.00245\times RH,\;R^{2}=0.9806$$

**Table A1:** Estimated parameters of *D*_eff_.

Drying Conditions	*D*_o_/m^2^·s^−1^	a	b	*E*_a_/kJ·mol^−1^	*R* ^2^	*RMSE*
35 °C RH60%	3.74 × 10^−7^	0.92	0.196	18.65	0.991	0.028
40 °C RH60%	3.74 × 10^−7^	0.81	0.179	18.53	0.991	0.028
45 °C RH60%	3.74 × 10^−7^	0.68	0.160	18.31	0.988	0.031
35 °C RH40%	3.74 × 10^−7^	0.52	0.147	17.06	0.999	0.009
35 °C RH50%	3.74 × 10^−7^	0.67	0.166	17.90	0.996	0.019
